# Non-alcoholic fatty liver disease

**DOI:** 10.1186/s12916-017-0806-8

**Published:** 2017-02-28

**Authors:** Brent A. Neuschwander-Tetri

**Affiliations:** Saint Louis University, 3635 Vista Ave, GI FDT-9, St. Louis, MO 63110 USA

**Keywords:** Non-alcoholic steatohepatitis, Lipotoxicity, De novo lipogenesis, Fibrogenesis, Insulin resistance, Fatty acids

## Abstract

Non-alcoholic fatty liver disease has emerged a major challenge because of it prevalence, difficulties in diagnosis, complex pathogenesis, and lack of approved therapies. As the burden of hepatitis C abates over the next decade, non-alcoholic fatty liver disease will become the major form of chronic liver disease in adults and children and could become the leading indication for liver transplantation. This overview briefly summarizes the most recent data on the pathophysiology, diagnosis, and treatment of non-alcoholic fatty liver disease. Ongoing clinical trials are focused on an array of disease mechanisms and reviewed here are how these treatments fit into the current paradigm of substrate overload lipotoxic liver injury. Many of the approaches are directed at downstream events such as inflammation, injury and fibrogenesis. Addressing more proximal processes such as dysfunctional satiety mechanisms and inappropriately parsimonious energy dissipation are potential therapeutic opportunities that if successfully understood and exploited would not only address fatty liver disease but also the other components of the metabolic syndrome such as obesity, diabetes and dyslipidemia.

## Background

Non-alcoholic fatty liver disease (NAFLD) has become increasingly common in parallel with the increasing prevalence of obesity and other components of the metabolic syndrome [1, 2] and it is projected to be the leading indication for liver transplant within a decade [3]. Briefly reviewed here are key studies presented in the past two years that have shed light on the natural history, diagnosis, and pathogenesis of non-alcoholic steatohepatitis (NASH). Data from recent treatment trials are reviewed and placed in the context of our current understanding of the pathogenesis of NASH.

## Natural history

NASH clearly progresses to cirrhosis with further decompensation leading to death or liver transplantation in some individuals. Unfortunately we still do not have a firm handle on how often this occurs based on longitudinal studies, but the estimates based on cross-sectional data are that 20-30% of adults living in affluent parts of the world consuming a western diet have too much fat in the liver (i.e., NAFLD), 2-5% have the subset of NAFLD in which substantial liver injury is also present (i.e., NASH) and 1-2% of all adults may be at risk for progressing to NASH cirrhosis [4]. The projected annual economic impact of this disease burden has been estimated to be $103 billion in the US and €35 billion in the UK, Germany, France, and Italy combined [5].

An ability to identify which patients are at greatest risk for progressing to cirrhosis is essential for targeting therapeutic interventions. Several studies have demonstrated the importance of any degree of liver fibrosis in the setting of NAFLD in predicting adverse outcomes. The late Paul Angulo and his coauthors collected data on 619 patients who had repeated liver biopsies (median 12.6 years apart) across multiple continents and reassessed their biopsies by one expert pathologist [6]. They demonstrated that fibrosis, hepatocyte ballooning and portal inflammation but not steatosis correlated with reduced survival. Loomba and colleagues also examined outcomes and demonstrated that fibrosis progression does occur in NAFL (NAFLD that is not NASH) but at a slower rate than in NASH [7].

Earlier studies have shown that the presence of type 2 diabetes (T2DM), obesity, and older age are associated with NASH and advanced fibrosis. A study by Wong and colleagues in Hong Kong using vibration controlled elastography (Fibroscan) identified increased liver stiffness in 17.7% of their diabetics and liver biopsies in a subset of their cohort identified NASH in 50% and stage 3-4 fibrosis in 57% [8]. A European population study similarly showed increased liver stiffness in 17.2% of people with T2DM and NAFLD [9].

**Fig. 1:**
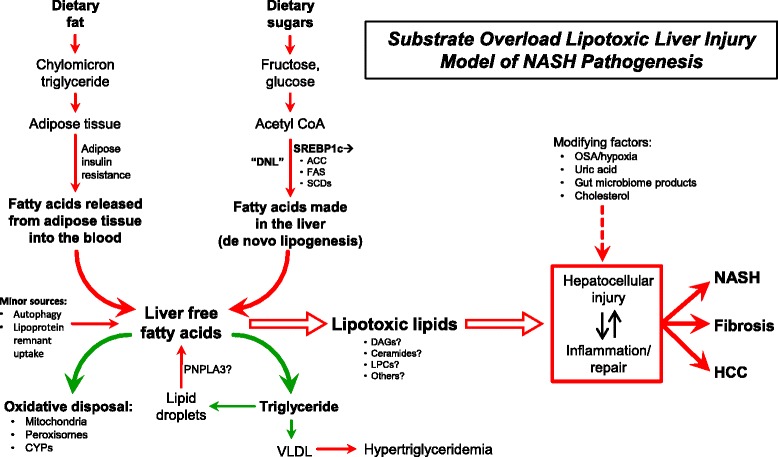
Substrate overload lipotoxic injury (SOLLI) model of NASH pathogenesis. The primary metabolic substrates are the monosaccharides glucose and fructose that are turned into fatty acids in the liver and fatty acids themselves that are delivered to the liver from adipose tissue. From this perspective, the most proximal abnormalities in the pathogenesis of NASH are the supply of excess dietary carbohydrates and fatty acids. The carbohydrates are derived from dietary intake and the fatty acids primary from adipose tissue, especially in the setting of insulin resistance. Carbohydrates can be converted to fatty acids through the multi-enzymes process of de novo lipogenesis and the transcription factor SREBP1c plays a dominant role in regulating the expression of these enzymes. Fatty acids in the liver can be oxidized by mitochondria or converted back into triglyceride for export into the blood as VLDL. In the setting of carbohydrate and fatty acid substrate overload or impairment of the pathways of fatty acid disposal, or perhaps most likely a combination of both arms, fatty acids may promote the generation of lipotoxic species (e.g., diacylglycerols [DAGs], ceramides, lysophosphatidyl choline species [LPCs]) that mediate endoplasmic reticulum stress, mitochondrial dysfunction, hepatocellular injury, inflammation, and apoptosis to produce the histological phenotype currently called NASH. These processes are then the stimuli for fibrogenesis and possibly malignant transformation. Major modulators of the hepatocellular response to lipotoxic stress may include the gut microbiome, a variety of cytokines, chemokines, and adipokines, free cholesterol, uric acid, free cholesterol and possibly periodic hypoxia caused by obstructive sleep apnea (OSA). DNL, de novo lipogenesis; SREBP1c, sterol response element binding protein-1c; ACC, acetyl-Coenzyme A carboxylase; FAS, fatty acid synthetase; SCD, stearoyl-Coenzyme A desaturase; CYP, cytochrome P450; PNPLA3, patatin like phospholipase domain containing 3; VLDL, very low density lipoprotein; OSA, obstructive sleep apnea; HCC, hepatocellular carcinoma

The observation that steatosis does not necessarily correlate with outcomes may seem unintuitive as we all tend to be impressed by substantial steatosis on imaging or liver biopsy. But when considered in the context of the pathophysiology of NASH (Fig. 1), the transient storage of fatty acids as inert triglyceride, the primary component of steatosis, may actually be an adaptive or protective mechanism rather than part of the pathophysiology and thus the magnitude of this accumulation may not be not directly related to liver injury. That being said, examination of the NHANES dataset where liver histology is not known demonstrated that severe steatosis by ultrasound or an elevated alanine aminotransferase (ALT) are both associated with increased liver related mortality [10]. How much of this risk is driven by undiagnosed NASH is unknown.

NAFLD is also associated with hepatocellular carcinoma (HCC) [11]. HCC is commonly thought of as occurring in the setting of cirrhosis or after decades of chronic hepatitis B infection, and only occasionally in the setting of chronic liver disease that has not yet progressed to cirrhosis. Importantly, a recent survey of US veterans demonstrated that the preponderance of non-cirrhotic HCC occurs in patients with NAFLD compared to other causes of chronic liver disease [12]. This observation suggests that we may need to rethink the strategy of confining our current practice of performing surveillance imaging only in cirrhotic NASH patients.

## Diagnosis

Whereas NAFLD can be diagnosed by imaging studies such as ultrasound, computed tomography (CT), or magnetic resonance imaging (MRI), the presence of NASH still requires a liver biopsy to identify the presence and location of its features such as inflammation, hepatocyte ballooning, Mallory-Denk bodies, and early fibrosis [13]. Because of the invasive nature and cost of a biopsy, non-invasive means of detecting NASH and various stages of liver fibrosis are sorely needed. A study measuring specific serum metabolites identified by mass spectrometry plus the presence of elevated AST, fasting insulin and the *PNPLA3* genotype was found to be good at discerning NASH from NAFL in a northern European cohort [14]. NASH is primarily a disorder of fat metabolism and thus serum lipidomic studies may offer the best opportunity to find specific lipids in the blood that can distinguish NASH from NAFL. Using a purely lipidomic approach, Loomba and colleagues found specific oxidized arachidonic acid species that robustly differentiated NASH from NAFL in a small but extensively characterized cohort in the US [15].

Non-invasively assessing fibrosis is the other major unmet need in NASH diagnostics. A large number of algorithms based on clinical data and imaging to assess fibrosis have been developed, but their major strength tends to be in identifying advanced fibrosis with less utility in earlier stages [16–19]. A newer technique takes a different approach by looking at collagen turnover using stable isotope labeling of new collagen [20], a technique that may have promise in treatment trials where current histological, serum and instrument based testing lack sensitivity for small changes over short time periods.

## Pathogenesis

Data from animal and human studies supports the concept that the hepatocellular injury that characterizes NASH is driven by an overload of primary metabolic substrates (glucose, fructose and fatty acids) in the liver resulting in diversion of fatty acids into pathways that promote cellular injury and a dysfunctional response to that injury (Fig. 1) [21–26]. Different aspects of these pathways leading to NASH and the resulting fibrosis likely vary among patients similar to the associated complex diseases obesity and diabetes [27].

## Treatment

**Fig. 2:**
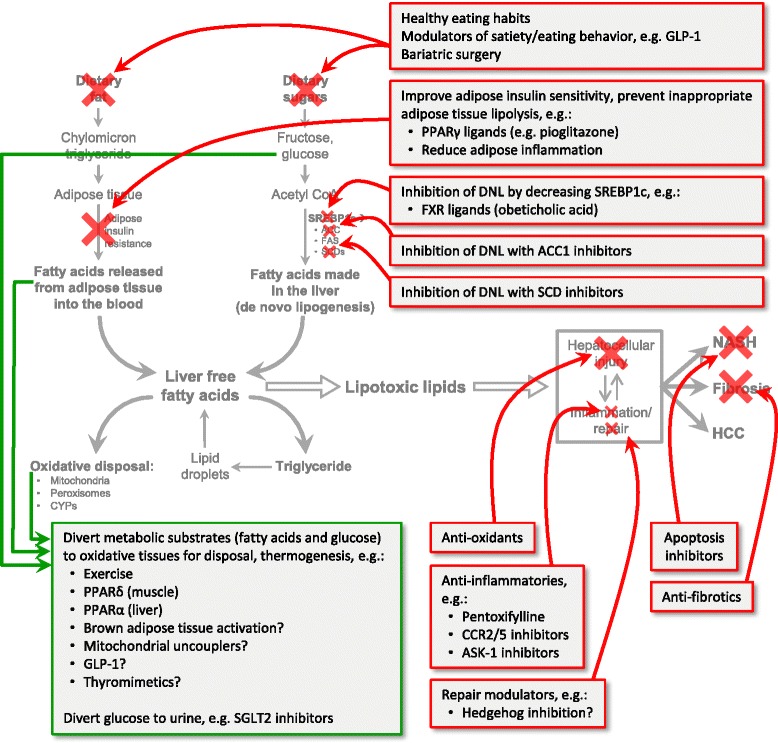
The SOLLI model predicts targets of therapy. Shown are many of the agents that have been studied in recent clinical trials or are the subject of ongoing trials. Healthy eating habits and bariatric surgery regulate the intake of metabolic substrates and thus their reduction is a treatment approach targeting the most proximal events in the process. Pharmacologic manipulation of eating behaviors and satiety may also be effective proximal interventions. Adipose tissue insulin resistance allows inappropriate lipolysis and release of fatty acids into the circulation which can be taken up by the liver. Both fatty acids and glucose in the blood can be diverted to oxidative pathways (green arrows) in other tissues and these pathways are thought to be augmented by exercise, PPARγ and PPARδ ligands, GLP-1 analogues, and other hypothetical interventions under investigation. The synthesis of fatty acids in the liver, or de novo lipogenesis, can be down-regulated by decreasing the regulatory transcription factor SREBP1c or by inhibiting specific enzymes in the DNL pathway. Fatty acids in the liver can be used in a large number of metabolic pathways but for disposal, they are oxidized by mitochondria, peroxisomes, and certain cytochrome P450 isoforms (CYPs) or reesterified to glycerol to form triglyceride. Pharmacologic promotion of triglyceride formation would increase lipoprotein secretion into the blood as very low density lipoprotein (VLDL) and could thus increase the risk of cardiovascular disease--not a likely treatment approach for NASH. Little is known about the lipotoxic species generated in NASH, but once these are better characterized, specifically inhibiting their formation or accelerating their disposal could become effective treatment approaches. Many of the treatment approaches in current clinical trials are focused on managing the consequences of lipotoxic injury by using anti-inflammatory agents, anti-apoptotic agents and anti-fibrotics. (Red arrows indicate inhibitory approaches; green arrows indicate possible beneficial diversion of metabolic substrates)

Lifestyle modification with a focus on healthy eating, weight loss when needed, and regular exercise remain the cornerstone of therapy in adults [28–31] and children [32]. When recommending healthy food choices, a Mediterranean diet has been shown to be a good alternative to a western diet [13, 33]. Bariatric surgery can be a good option in selected patients and a long term follow up study has been shown to reverse NASH and even substantial fibrosis in some [34, 35]. However, surgery is possible in only a minority of patients and there is clearly a need for pharmacological therapy [36, 37]. Prior clinical trial data suggest that pioglitazone or vitamin E may be beneficial in non-diabetic NASH patients [38] and the benefit of pioglitazone on reversing NASH and improving fibrosis was recently confirmed in diabetic patients [39]. More recent trial results are reviewed below and the substrate overload lipotoxic liver injury (SOLLI) model of NASH pathogenesis provides an organized approach to understanding these multiple potential points of attack (Fig. 2).

There are no approved drugs for NASH but recent trial data suggests that different approaches may be beneficial in subgroups of patients with NASH. It probably makes sense that no single therapy will reverse NASH in all patients since different patients likely manifest the phenotype of NASH in response to different genetic predispositions and environmental exposures. In addition, a major challenge for taking potential treatments through to approval by government agencies has been identifying meaningful trial endpoints. The field has moved forward due to the combined efforts to address these issues by regulatory agencies, industry, and academics [40].

The peroxisomal proliferator activated receptor (PPAR) family of nuclear receptors sense the presence of lipophilic molecules and regulate gene expression accordingly. PPARα upregulates oxidative metabolism in the liver and PPARδ does so predominately in muscle. The PPARα/δ ligand elafibranor was evaluated in the GOLDEN trial and appeared to resolve NASH in the subgroup with more severe disease at baseline who also received the highest dose [41]. Fibrosis improvement was also found in those whose NASH resolved. These findings have led to the initiation of an ongoing phase 3 trial.

In a different approach to modulating metabolism, a bile-acid derived ligand for the nuclear hormone receptor FXR, obeticholic acid, was evaluated in the FLINT trial. This drug was recently approved to treat primary biliary cholangitis at a dose of 10 mg daily and in the FLINT trial, subjects received a higher dose of 25 mg daily for 72 weeks resulting in improvements in the composite NAFLD activity score and fibrosis [42]. This drug is also in a phase 3 trial.

Modulating the glucagon-like-1 (GLP-1) incretin pathway has been a valuable adjunct in the treatment of type 2 diabetes because of the diverse favorable effects of GLP-1 and its analogues in modulating metabolism at multiple targets in the body. The GLP-1 analogue liraglutide is used to treat diabetes and was evaluated in patients with NASH in the LEAN trial in the UK [43]. It was a small trial and barely met the primary endpoint of resolution of NASH without worsening of fibrosis (9/23 vs 8/22 placebo treated patients, P 0.019). In a study of sitagliptin, a drug that prevents breakdown of endogenous GLP-1, the drug was not found to have any effect on liver histology or ALT in patients with NASH [44]. Thus augmenting the GLP-1 axis may have an adjunctive role in the context of combination therapy but is unlikely to play a major role as monotherapy.

Based on the hypothesis that the lobular inflammation in NASH contributes to hepatocellular injury and fibrogenesis [45], anti-inflammatory agents have been investigated. A prior study of pentoxifylline was promising [46] but a trial of an effective anti-inflammatory phosphodiesterase-4 inhibitor was negative [47]. More recently, an antagonist of the chemokine 2 and chemokine 5 receptors (CCR2/5) called cenicriviroc has been evaluated and the preliminary trial results suggest improvements in fibrosis [48].

Even if treatments are found that reverse NASH, some patients will continue to present with advanced fibrosis or cirrhosis and thus effective antifibrotic agents may always be needed [30]. Effective anti-NASH drugs will likely have indirect antifibrotic effects by eliminating the stimulus for fibrogenesis, but some drugs have been designed to be directly antifibrotic (e.g., the galectin-3 inhibitor MD-02) or increasing extracellular matrix turnover (e.g. simtuzumab) and are currently in clinical trials.

## Future directions

Many of the current pharmacological approaches to treating NASH are focused on relatively downstream events of liver injury, inflammation and fibrogenesis. It may be advantageous to manipulate the upstream events leading to substrate overload such as central nervous system control of satiety mechanisms and energy efficiency [49, 50]. Recent insights into the genetic control of eating-related reward signaling [51] and peripheral input to the CNS modulating the central control of metabolism [52] raise the possibility of developing therapeutics not only for the liver but the other components of the metabolic syndrome.

## Conclusions

As we enter an era of increasing genomic, lipidomic and metabolomic information, the future is bright for improving our understanding of the pathogenesis of NASH to the point where we can provide individualized treatment. A challenge in the field now is to correlate the emerging data with treatment responses to attain this goal.
